# Significant improvement of spray pyrolyzed ZnO thin film by precursor optimization for high mobility thin film transistors

**DOI:** 10.1038/s41598-020-65938-6

**Published:** 2020-06-02

**Authors:** Jewel Kumer Saha, Ravindra Naik Bukke, Narendra Naik Mude, Jin Jang

**Affiliations:** Advanced Display Research Center (ADRC), Department of Information Display, Kyung Hee University, 26, Kyungheedae-ro, Dongdaemun-gu, Seoul 02447 Korea

**Keywords:** Engineering, Materials science

## Abstract

Metal-oxide thin-film transistors (TFT) fabricated by spray pyrolysis are of increasing interest because of its simple process and scalability. A bottleneck issue is to get a bubble-free and dense material. We studied the effect of ammonium acetate (AA) addition in the oxide precursor solution on the performance of spray-coated ZnO TFTs. AA acts as a stabilizer, which increases the solubility of the solution and enhances the film quality by reducing the defects. With AA addition in ZnO precursor, the films are coffee ring free with high mass density and better grain orientation. The ZnO TFT with AA exhibit a remarkable improvement of its device performance such as saturation mobility increasing from 5.12 to 41.53 cm^2^V^−1^s^−1^, the subthreshold swing decreasing from 340 to 162 mV/dec and on/off current ratio increasing from ~10^5^ to 10^8^. Additionally, the TFTs show excellent stability with a low threshold voltage shift of 0.1 V under gate bias stress. Therefore, the addition of AA is a promising approach to achieve high-performance ZnO TFTs for low-cost manufacturing of displays.

## Introduction

The demand for cutting-edge, low voltage and high-performance thin-film transistors (TFTs) drives the research towards high-mobility oxide semiconductors for high-definition displays. Nowadays, the majority of TFTs used in the products are based on Si thin film, mainly amorphous silicon (a-Si) and polycrystalline silicon (poly-Si) on the glass. The a-Si shows low mobility (<1 cm^2^V^−1^s^−1^)^1^, and poor transparency in the visible range due to its narrow bandgap. The metal oxides are expected to substitute Si thin film in several applications^2–4^. Among the various metal oxides, zinc oxide (ZnO) has attracted much attention due to its wide bandgap (~3.37 eV), nontoxic nature, low cost, structural tunability, optical and electrical properties^5^. Pure ZnO TFTs fabricated by radio frequency magnetron sputtering show the mobility up to 70 cm^2^V^−1^s^−1 ^^6^. ZnO thin films can be deposited by several techniques such as sputtering, chemical vapor deposition, pulsed laser deposition, spin coating, spray pyrolysis, etc^7–15^. Among these techniques, spray pyrolysis is a simple and low-cost vacuum-free deposition method to produce reliable and uniform films on large scale^16^. Deposition by spray-pyrolysis produces a uniform and reproducible metal-oxide-semiconductor films, which can cover a large area compared to ink-jet printing and/or spin coating. Meticulous control of the deposition parameters such as flow rate, the distance between the substrate holder (hot plate) and nozzle, deposition temperature, and the speed of the nozzle movement, can improve the quality of the film.

Many groups report ZnO TFTs fabricated by spray pyrolysis (SP) technique. The main challenge of spray coating is to make bubble-free, dense thin films. During the spray pyrolysis, the quick evaporation of the solvent often results in bubble rings or/and pinholes throughout the film. Ivan *et al*. reported the Leidenfrost effect forming coffee rings during spray on the hot plate^17^. The coffee rings result in a film due to irregular orientations of grains, which are the origin of reducing the carrier mobility of the devices. Note that the highest mobility of 32 cm^2^V^−1^s^−1^, is reported so far in spray deposited pure ZnO films^40^. In this work, we studied the effect of adding ammonium acetate (AA) in the ZnO precursor solution for ZnO film deposited by spray pyrolysis at 350^o^C and used for high-performance TFTs. AA is added as a stabilizer in the precursor solution. It increases the solubility of the solution and the excess of acetate groups (confirmed by NMR) reduces the evaporation rate of the solvent during spray. This increases the diffusion length of droplets, resulting in very thin, uniform, bubble/ pinhole-free ZnO films. The improved quality of the ZnO films such as smoothness, compactness and defect concentration is confirmed by atomic force microscopy (AFM), scanning electron microscopy (SEM) and X-ray photoelectron spectroscopy (XPS). Finally, we show that the device performance of the ZnO TFTs such as mobility, subthreshold swing (SS), the threshold voltage (V_TH_) and on/off current ratio are remarkably improved with the addition of the AA.

## Results and Discussion

**Figure 1:**
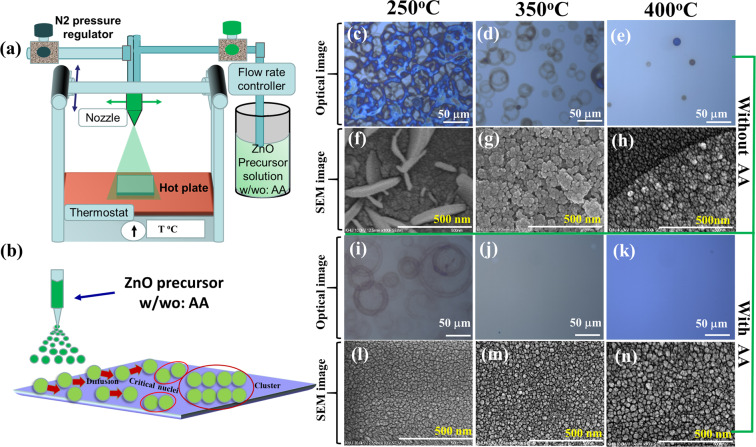
Nucleation and surface morphology of ZnO thin film without and with ammonium acetate (AA) deposited by spray pyrolysis at substrate temperature of 250, 350, and 400 ^o^C. Schematic of **(a)** the spray system used for the experiment and **(b)** the nucleation process at the time of pyrolysis. Optical microscopy and SEM image of ZnO thin films deposited at 250, 350, and 400 ^o^C **(c–h)** without and **(i–n)** with AA in precursor solution. After direct impingement of droplet on the hot substrate, the droplet changes the phase from vapor to solid and results unwanted coffee rings. The presence of AA in precursor solution helps the droplet to levitate on the substrate and increase the diffusion length on the substrate. As a result, coffee rings free uniform film with high compactness are found by slow evaporation of solvents.

The ZnO thin films were deposited on glass by spray pyrolysis using precursor solutions without and with AA. Figure 1a shows the schematic diagram of the spray system. The deposition temperature was varied from 200 to 400 ^o^C, the flow rate (3 ml/min) and the nozzle speed (8 cm/sec) were kept constant to find the optimum temperature. Figure 1b shows the schematics of the nucleation and growth process. There are four forces of gravitational, electrical, stokes and thermophoretic forces, which push the droplets away from a hot surface. When a droplet is approaching the hot substrate, craters are generated immediately due to the fast flow of hot air. As a result, the droplets levitate on the surface and are deposited to form the film with solvent evaporation. When the monomer is deposited on the hot substrate, it diffuses on the substrate until it re-evaporates or incorporates into a stable nucleus, which further grows into the larger grain. If the diffusion length of the incoming monomers is long enough, the growth can occur laterally (i.e. ~ bidimensional growth). The diffusion of the monomer depends on the substrate temperature, the surface energy of the film, the binding energy between the film and substrate, the boiling point of the precursor solution and the deposition rate^18^. When the droplet loses its normal velocity component and is adsorbed on the substrate, tri-dimensional growth takes place. This direct impingement of the spray droplet from the vapor phase results in unwanted coffee rings or bubbles in the film. Isakov *et al*.^19^ reported the nucleation boiling, transition boiling, and Leidenfrost boiling points for the respective temperature ranges of T < 200 °C, 200 °C < T < 250 °C and T > 250 °C respectively. For temperatures over 250 °C, the droplets are levitating due to the apparition of the thin gaseous layer between the substrate and the droplets. This results in higher effective diffusion length for the precursors, leading to the lateral growth of the film and reduces the density of bubbles.

The ZnO precursor solutions without and with AA were sprayed from low temperatures (200 °C) to high temperatures (400 °C). Figure 1(c–h) shows the optical microscopy and SEM images of ZnO films without AA deposited on glass at 250, 350, and 400 °C, respectively. As the temperature increases, the density and the size of the coffee rings/bubbles decrease. As shown by SEM in Fig. 1(f–h), the films formed at 250 °C exhibit a mixture of large rods and islands, at 350 ^o^C only islands are formed with an apparent decrease of the overall roughness. The rods might form due to the limited diffusion length at low temperatures, resulting in substrate plane nucleation and growth. At temperatures over 300 °C, the diffusion length becomes high enough for lateral growth. Figure 1(i–n) shows the optical and SEM images of ZnO films deposited from the precursor solution containing AA at the substrate temperature from 250 °C to 400 °C. At 250 °C, the density of bubbles is much lower than that one in the film sprayed without AA. Moreover, at ≥350 C the films do not show any bubble. The SEM images in Fig. 1(l–n) show that films with AA consist of small islands and no rods are found. The inhomogeneous nucleation at the coffee ring boundaries would be due to the direct impingement of droplets. The diameter of one ring is around 20 μm and that its boundary is decorated with large rods (See Supplementary Fig. S1(a,b)). As the solvent evaporates, the solutes are gathering at the boundary and vertical growth is dominating during nucleation. The presence of AA at high substrate temperature increases the diffusion length and slow evaporation makes the film uniform and compact. We can see the effect of temperature and AA on the morphology of the ZnO film (See Supplementary Fig. S1(c,d)). Additional optical and SEM images for films grown at 200 °C and 400 °C are shown (Supplementary Fig. S2). This suggests that adding AA to the precursor solution increases the diffusion length of the Zn precursors on the growing surface during spray coating.

**Figure 2:**
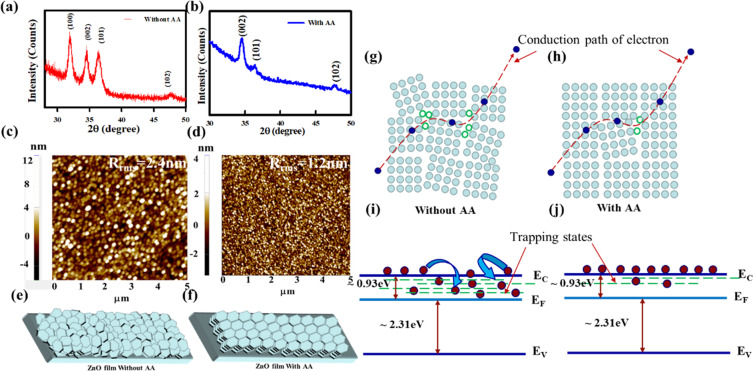
Structural properties and surface morphology of ZnO thin film without and with AA.XRD spectra, AFM image and schematic of crystallization of the ZnO thin film on glass substrate are shown in **(a,c,e)**, respectively, for precursor solution without AA and **(b,d,f)** for precursor solution with AA. (**a**) shows the three peaks at 2θ of 31.9°, 34.5°, and 36.4° corresponding respectively to (100), (002) and (101) planes of the hexagonal ZnO wurtzite structure. The intensity of the (100) and (101) peaks decreases significantly with the addition of AA, shown in (**b**), which indicates a c-axis aligned crystallization (CAAC) of the ZnO film. With adding AA in the precursor solution, ZnO thin film shows no bubbles, and the roughness of the film decreases from 2.4 to 1.2 nm. **(g–j)** Representation of conduction path of electron at grains and grain boundary in the ZnO film with a model of energy band diagram showing trapping states for ZnO device following **(g,i)** without AA in precursor solution and **(h,j)** with AA, respectively.

We studied the morphology of thin films in more detail. Figure 2(a,b) shows the X-ray diffraction pattern of the crystalline ZnO thin films deposited by spray pyrolysis at 350 °C without and with AA, respectively. The three peaks at 2θ of 31.9°, 34.5°, and 36.4° correspond respectively to the (100), (002) and (101) planes of the hexagonal ZnO wurtzite structure. With the addition of AA, the intensity of the (100) and (101) peaks decreases significantly, which indicates a strong texturing of the films along the (002) c-axis. This suggests that the grains observed by SEM in Fig. 1g (i.e. without AA) are randomly oriented, while the ones in Fig. 1m (i.e. with AA) are all aligned along the c-axis. The surface morphology of the ZnO thin films was studied by atomic force microscopy (AFM). Figure 2(c,d) show the AFM images of the surfaces of the films spray-coated without and with the addition of AA respectively. The RMS roughness of the ZnO film is found to decrease from 2.4 nm to 1.2 nm with the addition of AA. As schematically shown in Fig. 2e, the random orientation of the grains (without AA) tends to produce rough surface, while when the grains are well aligned along the c-axis grains (with AA) the film has a smooth surface as shown in Fig. 2f.

In nanocrystalline ZnO, the electron trapping sites at grain boundaries control electron conduction^20–24^. Fig. 2(g,h) schematically shows the conduction path of electrons in grains and at grain boundaries for randomly oriented grains in the film grown without AA (Fig. 2g) and for the uniformly oriented grains for the ZnO layer grown with AA (Fig. 2h). The latter film has fewer defects and trapping sites. The energy band diagrams of Fig. 2(i,j) illustrate that the reduction of the trapping state density for the film using the ZnO grown with AA.

**Figure 3:**
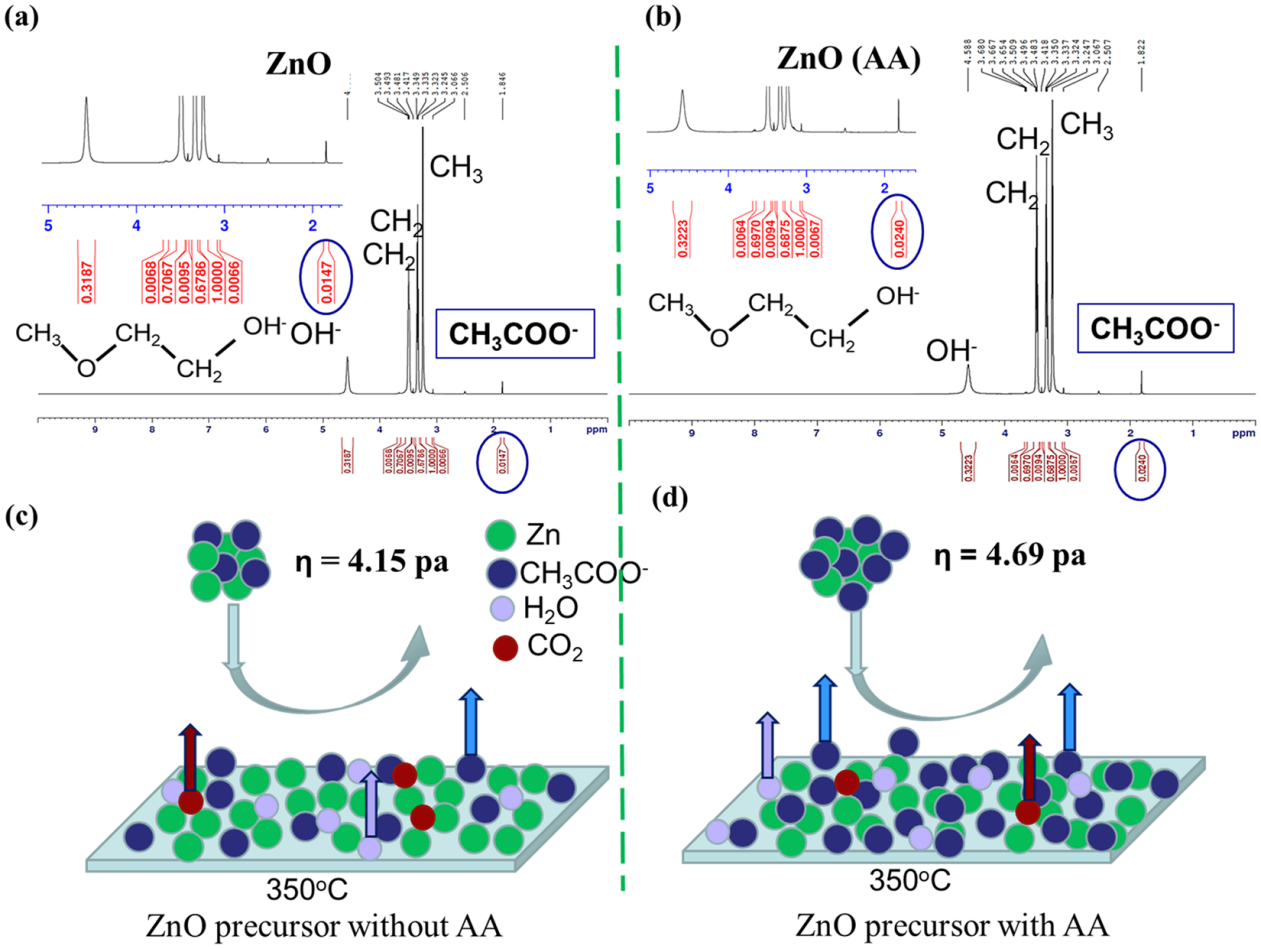
Quantitative study of concentration of different molecules and mixture of complex. present in ZnO solution without and with AA. **(a,b)** 1 H NMR spectra of ZnO precursor solution without **(a**, left**)** and with ammonium acetate **(b**, right**)**. The presence of more acetate groups in the ZnO precursor solution with AA makes the film smoother and uniform. **(c,d)** Schematic of solvent evaporation during spray pyrolysis for precursor solution **(c)** without and **(d)** with AA. In Figure **(c,d)**, ƞ is the viscosity of the ZnO solution.

To get more insight into the effect of ammonium acetate in the precursor solution, we checked the NMR (Nuclear Magnetic Resonance) spectra of both precursor solutions without and with AA. Figure 3(a,b) shows the 1 H NMR spectra of ZnO precursor solutions without (left) and with AA (right). We did not find any trace NH^3+^ (at 5.3ppm), which indicates that there is no [Zn (NH_3_)_4_]^2+^ complex in the solution. This suggests that all the ammonia evaporates from the precursor solution during the preparation. Thus, ammonia does not contribute to the ZnO thin film formation and morphology improvement. However, we found the presence of more acetate in the ZnO solution with AA. The integral of the CH_3_COO^−^ intensity is 0.0240 for precursor with AA, which is the double of 0.0147 for precursor without AA. Consequently, the addition of AA increases the acetate group in the precursor solution. The increase of the acetate group enhances the viscosity (ƞ) of the solution from 4.15pa without AA to 4.69pa with AA. During spray pyrolysis, the excess of the acetate group slows down the evaporation rate and increases the diffusion length of the droplet on the hot substrate. This results in smooth and uniform thin films with well-oriented grains with no bubbles, as shown in Fig. 3(c,d). The density of ZnO film with AA is 5.18 g/cm^3^, which was confirmed by XRR (X-Ray Reflectivity) (see Supplementary Fig. S3). Note that the density of crystalline ZnO is 5.6 g/cm^3 ^^25^.

Figure S4a shows the UV-Vis transmittance spectra of ZnO films without and with AA. The inset in the Figure depicts a photograph of the Advanced Display Research Center (ADRC) logo under the ZnO thin film. The ZnO film with AA shows 90% transmittance, which is higher than that of the film without AA (84%) in the visible range. The improved transmittance originates from the better alignment of the grains in the film, reducing the light scattering. This low-cost spray coating method for ZnO with AA can be favorably used for transparent electronics^26^.

The optical band gap can be extracted for the transmission data using the following relation: αhν = A (hν-E_g_)^1/n ^^27^, where hν is the photon energy, A is a proportionality constant, E_g_ is the optical bandgap of the semiconductor and α the absorption coefficient. The exponent ‘n’ is related to the nature of the optical transitions involved in the absorption process and it is 1/2 in the case of direct optical transitions in ZnO. We found a value 3.24 eV for the optical bandgaps of both without and with AA films, in good agreement with the reported values of ~3.3 eV^2^.

In-depth profile X-ray photoelectron spectroscopy (XPS) analysis reveals the chemical states of the elements and their distribution in the film. Here, the ZnO film was deposited on an AlO_x_ layer on the glass substrate to mimic the TFT structure. Wide-scan spectra (on the surface) and depth profile of ZnO film with AA are shown in Fig. S5(a,b), respectively. At the surface of the ZnO film, the existence of XPS peaks of Zn 2p and O 1 s confirms the formation ZnO. Additionally, the ZnO and AlO_x_ layers are confirmed from the depth profile. Furthermore, there is no Al in ZnO film, which confirms the absence of Al diffusion into ZnO film. We found C atoms at the ZnO surface, but not in the bulk. The presence of C at the surface is due to contamination during XPS measurement (particularly during etching the film from surface to depth). The carbon usually acts as a carrier-trapping site^25^. From the XPS depth profile, at the surface of ZnO film we found that Zn, O, and C with 40.41, 42.53, and 17.06 at.%, respectively. Whereas, only Zn, O, and C are found at the bulk with 58.57, 41.43, and 0 at.%, respectively.

**Figure 4:**
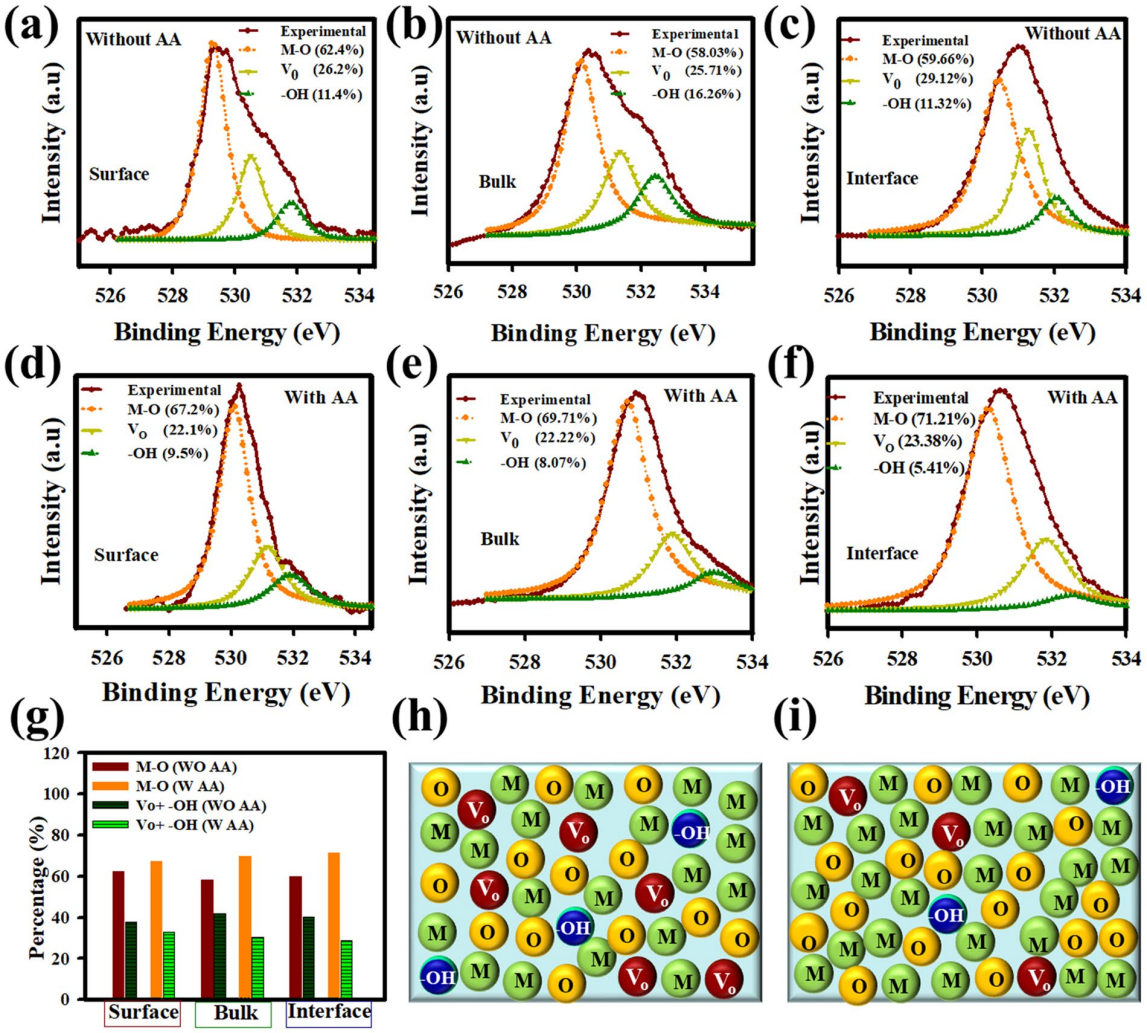
XPS spectra analysis of deconvoluted O1s peak at the surface, bulk and ZnO/ AlO_x_ interface without and with AA. **(a–c)** Deconvoluted O1s peak at the surface, bulk, and ZnO/ AlO_x_ film, respectively for ZnO without AA. **(d–f)** Deconvoluted O1s peak at the surface, bulk, and ZnO/ AlO_x_ interface, respectively for ZnO with AA. The individual contribution of metal oxide (M–O), oxygen vacancy (V_o_) and metal hydroxyl group (M−OH) are shown with orange curve (~529.5 eV), dark yellow (~531 eV), and green curve (~532 eV), respectively. Due to AA the V_o_ and –OH are reduced at the surface, bulk and ZnO/AlO_x_ interface which leads the improvement of film quality as well as device performance. **(g)** The relative weight of the M–O and the defect (V_o_ + −OH) percentages are shown in the histogram. **(h,i)** The schematic of the proportion of metal (M), oxygen (O), oxygen vacancy (V_o_), and hydroxyl group (−OH) at the ZnO/AlO_x_ interface without and with AA in ZnO precursor, respectively. Here, ZnO film was deposited by spray pyrolysis at 350 ^o^C, and AlO_x_ film was spin-coated and annealed at 350 ^o^C in furnace.

In general, the carrier transport properties in ZnO are closely related to the chemical states of the elements in the ZnO, such as the metal-oxygen bond (M-O), oxygen vacancies (V_o_), and hydroxyl groups (-OH). Figure 4(a–f) shows the details of the O1s peaks deconvoluted using the Gaussian-Lorentz fitting method. Each peak is deconvoluted into three peaks at 530 eV, 531 eV, and 532 eV corresponding to M-O (Zn atoms surrounded by O^2−^ in a wurtzite structure), V_o_ (oxygen vacancies), and M-OH (hydroxyl groups), respectively. Figure 4(a–c) shows the deconvoluted O1s peak intensity at the surface of the ZnO film without AA, in the bulk, and the ZnO/AlO_x_ interface. Whereas, Fig. 4(d–f) show the deconvoluted O1s peaks at the same positions for ZnO films with AA. The relative weight of the M-O peaks and the defect (V_o_ + -OH) peaks are reported in the histograms of Fig. 4g. The surfaces of the films without and with AA show a similar proportion of M-O bonds and defects. However, in the bulk and at the AlO_x_ interface, the amount of M-O bonds increases while the density of defects decreases in the film with AA, compared with the film without AA. The increase of M-O bonding and the reduction in V_o_ and –OH groups are related to the improvement of the film quality^28–35^, The ZnO film without AA shows a higher percentage of the hydroxide group (12%) compared to the ZnO film with AA (6%). Oxygen vacancies are related to point defects in the crystalline ZnO film. It reduces from 29 to 23% by adding AA, which indicates that the point defects are reduced. Figure 4(h,i) shows the schematic of the proportion of metal (M), oxygen (O), defects due to oxygen vacancy (V_o_) and hydroxyl group (-OH) at the ZnO/AlO_x_ interface without and with AA in ZnO precursor, respectively. Those observations are consistent with the better alignment of the grains (reduced grain boundaries) and the higher mass density with AA.

Figure S6 shows the optical images of the ZnO TFT (a) without the addition of AA, and (b) with AA in the precursor. Clear active islands of the ZnO TFT without and with AA, are shown in Fig. S6. Figure S6 illustrates the presence of coffee rings on the active island for films without and with AA. Figure S7a shows the cross-sectional Transmission Electron Microscopy (TEM) micrographs and the Fast Fourier Transform (FFT) of a ZnO film with AA. The clear diffraction pattern in the FFT confirms the high crystallinity of the film and shows the alignment along the c-axis. The atomic rows in the film can be seen in detail from the high-resolution images of Fig. S7(b,c). The thickness of the ZnO film is ~20 nm. Only a single crystalline structure can be seen from the HRTEM image, with no grain boundaries or dislocations, which indicates the defect density in the film is very low.

**Figure 5:**
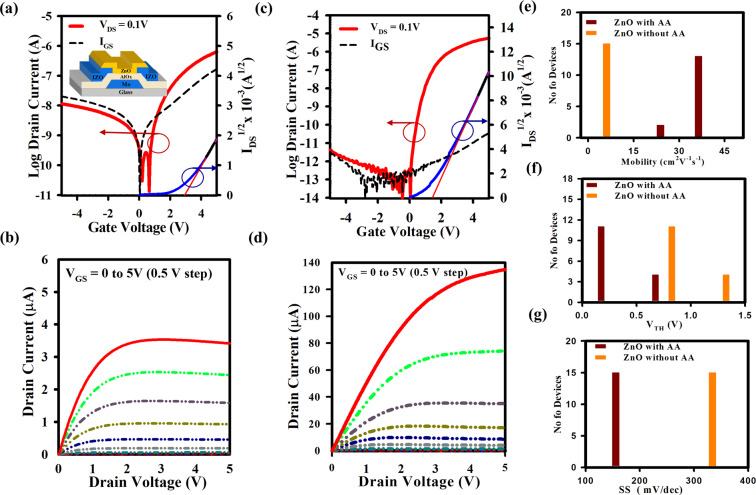
Electrical performance of ZnO TFT with and without ammonium acetate (AA). Plots of the **(a)** transfer characteristics, gate leakage current, and the square root of the drain current as a function of gate voltage, **(b)** output curves of ZnO TFT without AA. **(c)** Transfer characteristics, gate leakage current, and square root of the drain current as a function of the gate voltage, **(d)** output curves of ZnO TFT fabricated with AA. The transfer curve of the ZnO TFT were measured by sweeping V_GS_ from −5 to +5 V at the drain voltage, V_DS_ = 0.1 V. Output curve was measured by varying V_GS_ from 0 to 5 V with step = 0.5 V. **(e–g)** Performance summary of 15 ZnO TFTs for mobility, V_TH_, and SS, respectively, fabricated without and with AA. The addition of AA improves mobility, reduces V_TH_ and SS. ZnO channel layer was deposited by spray pyrolysis at 350 ^o^C.

To investigate the charge transport properties of the n-type ZnO thin films without and with AA, we fabricated the bottom gate, bottom contact (BGBC) TFTs. The structure of the TFTs is Mo (40 nm)/AlO_x_ (40 nm)/IZO (100 nm)/ZnO (20 nm) and is illustrated in the inset of Fig. 5a. The Mo and IZO layers were deposited at 280 °C and room temperature, respectively, by sputtering. The AlO_x_ films were spin-coated and annealed in air at 350 °C for 2 h. The ZnO films were spray coated. The pattern was defined by photolithography. The TFTs exhibit better performance when the ZnO precursor with AA is deposited at 350 °C (See Supplementary Fig. S8). Thus, we choose to deposit all ZnO films at 350 °C to compare the performance of TFTs made without and with AA. The transfer and output curves are shown in Fig. 5(a–d) for ZnO films without and with AA, respectively. The TFTs were measured by V_DS_ = 0.1 V with a forward sweep from −5 to +5 V. The ZnO TFT without AA exhibits the maximum μ_sat_ of 5.12 cm^2^V^−1^s^−1^, V_th_ of 0.88 V, SS of 340 mV/dec, and I_on_/I_off_ ratio of ~10^5^. The TFT with AA exhibits the dramatic increases of the μ_sat_ to 41.53 cm^2^V^−1^s^−1^, and I_on_/I_off_ ratio to ~10^8^. The ZnO film without ammonium acetate (AA) has bubbles or coffee rings which could make the interfacial issue between the channel and gate insulator (GI). Therefore, it is hard to make correct pattern at the place of bubbles during the channel layer etching. Thus, GI is affected and leakage current (I_GS_) through the GI increases. The ZnO film without and with AA shows the n-type semiconducting behavior. By using AA in the ZnO precursor solution, there are no bubbles in the ZnO film. Therefore, there is no issue for channel and GI layer etch. Consequently, we have a clear active island and improved interface between channel and GI. As a result, we have good TFT performances for ZnO TFT with AA. Hence, the reference ZnO TFT without ammonium acetate is also non-ambipolar. In negative V_GS_ the off current (I_OFF_) is high due to high leakage current through the GI. The trap density (N_SS_) at the interface calculated from SS and found to be 3.82 × 10^12^ cm^-3^eV^-1^ and 1.39 × 10^12^ cm^-3^eV^-1^ for the TFTs without and with AA, respectively. The N_SS_ obtained from SS reduces significantly with the addition of AA in the ZnO precursor solution. This is in good agreement with the XPS results showing a reduction of the defect density at the interface with AA. The output current for ZnO TFT without AA degrades slightly but the TFT with AA shows a good saturation behavior. There is no current crowding in the low drain current region, showing a good ohmic contact between S/D and the channel layer. Also, the ZnO TFTs with AA show clear pinch-off. The electrical performance (saturation mobility (μ_sat_), subthreshold swing (SS), and current on/off ratio (I_ON_/I_OFF_)) of ZnO oxide TFTs reported in the literature fabricated with different deposition techniques are summarized in Table 1^36–43^. Our results show the highest mobility among the TFTs by spray-coatings, with 30% improvement compared to the highest μ_sat_ (32 cm^2^/Vs) reported^44^.

**Table 1:** An overview of the processing method and electrical performances (field-effect mobility, subthreshold swing, and current on/off ratio) of the solution-processed ZnO TFTs from the literature.

Active Layer	Process	Substrate Temp. (^o^C)	μ_sat_ [cm^2^V^−1^s^−1^]	SS [mV/dec]	I_on_/I_off_	Year^ref^
ZnO	SP	400	10.00	550	10^6^	2015^36^
ZnO	SC	140	3.20	200	10^7^	2016^37^
ZnO	ALD	160	13.30	190	10^8^	2016^38^
ZnO	SC	100	0.43	220	10^5^	2017^39^
ZnO	SP	400	32	—	10^5^	2011^40^
ZnO	SC	Base 350	7.65	—	10^6^	2011^41^
ZnO	SC	Base 450	14.70	—	10^6^	2011^41^
ZnO	SC	Acid 450	0.19	—	10^5^	2011^41^
ZnO	SC	300	0.45	730	10^6^	2013^42^
ZnO	SP	250	12.00	300	—	2013^43^
ZnO	SP	350	41.53	162	10^8^	This work

SC: Spin Coating, SP: Spray Coating, Temp.: temperature, ALD: Atomic layer deposition.

To check the uniformity of the spray-coated TFTs we measured the characteristics of 15 TFTs at different positions on 7.5 ×7.5 cm^2^ TFT backplane with all TFTs of W/L = 20μm/3μm. The histograms of Fig. 5(e–g) summarize the performance of the 15 ZnO TFTs without and with AA and the average values are shown in Table 2. The mean values of the mobility are μ_sat_ 5.35 ± 3.80 (w/o/ AA) and 39.26 ± 2.27 cm^2^V^−1^s^−1^ (w/ AA), of the V_th_ are 0.83 ± 0.06 (w/o/ AA) and 0.58 ± 0.04 V (w/ AA), and of the SS are 344.25 ± 10.98 (w/o/ AA) and 167.40 ± 9.22 mV/dec (w/ AA). These results confirm the excellent uniformity of our device and the improvement of performance upon the addition of the AA in the precursor solution. The improvement of the ZnO TFTs with AA is due to the reduced amount of interface traps at the ZnO/AlO_x_ interface^28^. The high mobility can be attributed to the high quality of ZnO film as well as the good interface between the insulator and the active layer. The better quality of the interface is due to better alignment of the grains in the film.

**Table 2:** Summary of electrical performances of 15 ZnO TFT’s by spray pyrolysis without and with AA in ZnO Precursor solution.

ZnO TFT	µ_sat_ (cm^2^V^−1^ s^−1^)	V_th_ (V)	SS (mV/dec)
15 TFT (without AA)	5.35 ± 3.80	0.83 ± 0.06	344.25 ± 10.98
15 TFT (With AA)	39.26 ± 2.27	0.58 ± 0.04	167.40 ± 9.22

We studied the effect of ammonium acetate (AA) on the performance of ZnO TFT fabricated by spray pyrolysis at 350 ^o^C. The significant improvement in the quality of the ZnO film such as smoothness, film density, and fewer defects carried out by the addition of AA. Therefore, the devices with AA exhibit high saturation mobility (μ_sat_) of 41.53 cm^2^V^−1^s^−1^, which represents an 8-fold improvement compared to devices made without AA.

**Figure 6:**
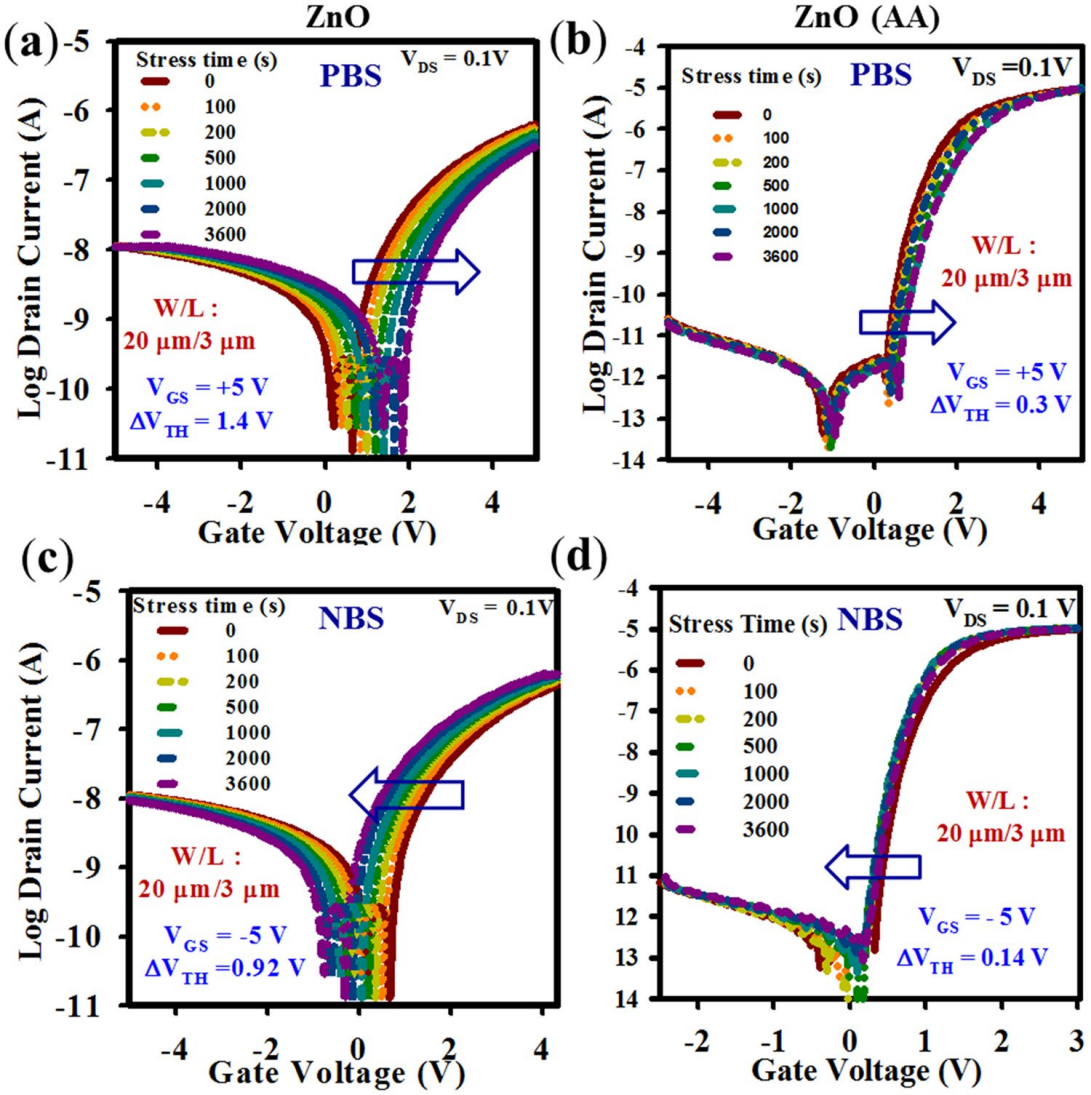
Bias stability of ZnO TFTs deposited by spray using the presursers without and with AA. The evolution of the transfer curves of ZnO TFT under PBS, were measured by sweeping the gate voltage from −5 to + 5 V at a constant drain voltage (V_DS_) of 0.1 V with V_GS_ of +5 V for 1 h for the TFTs **(a)** without and **(b)** with AA. NBS of ZnO TFT measured by sweeping the gate voltage from +5 to −5 V at a drain voltage (V_DS_) of 0.1 V with V_GS_ of −5 V for 1 h **(c)** without and **(d)** with AA for 1 h.

We have tested the electrical stability of the spray-coated ZnO TFTs without and with AA with positive bias stress (PBS) with V_GS_ = +5 V and negative bias stress (NBS) with V_GS_ = −5V for 1 h in dark at room temperature. The evolution of the transfer curves under PBS, measured by sweeping the gate voltage from −5 to +5 V at a constant drain voltage (V_DS_) of 0.1 V is shown in Fig. 6a w/o AA and Fig. 6b w/ AA, respectively. The evolution of the transfer curves under NBS, measured by sweeping gate voltage from −5 to +5 V and −3 to +3 V at the same V_DS_ of 0.1 V is shown in Fig. 6c for films w/o AA and Fig. 6d for films w/ AA, respectively. The threshold voltage shift (ΔV_TH_) under PBS for the ZnO TFT without AA is 1.4 V. On the other hand, the ΔV_TH_ shift for the ZnO TFT with AA is only 0.10 V. The positive shift of the transfer curve results from the trapping of electrons at the ZnO/AlO_x_ interface^28^. Thus, the small ΔV_TH_ of the TFT with AA again confirms the lower defect density at the interface between the gate dielectric and the active layer. Note that there is a negligible change in the SS after bias stress for 1 h, which indicates the presence of very few trapping sites at the interface^44^.

## Conclusion

We have studied the effect of the addition of ammonium acetate in the precursor on the morphology and TFT performance of spray-coated ZnO films. We show with AFM, SEM and XPS analysis that upon addition of AA, the overall quality of the ZnO films such as mass density, surface roughness, and crystalline structure is improved. XRD analysis shows a better texturing, i.e. a more uniform grain orientation in the films. The devices with AA exhibit high field-effect mobility of 41.53 cm^2^V^−1^s^−1^, which represents an 8-fold improvement compared to the TFT made without AA. This is also a 30% improvement compared to the highest values reported in the literature for spray coated ZnO TFTs. All the other characteristics of the TFTs are improved with addition of AA, the V_TH_ is 0.55 V, the SS is 162 mV/decade, and the I_ON_/I_OFF_ is 10^8^ at low operating voltage of 5 V. Moreover, TFT made with AA shows a better stability with a threshold voltage shift of 0.1 V under positive or negative gate bias stress. Therefore, this work contributes to the development of low operating voltage, high mobility ZnO TFT with excellent stability for the next-generation, low-cost displays.

## Experimental Section

### Precursor solution synthesis

Zinc acetate dihydrate (Zn(CH_3_COO)_2_ ·2H_2_O) and ammonium acetate (CH_3_CO_2_NH_4_) precursors from Sigma Aldrich, (99.999%) were dissolved into the solvent of 2-methoxyethanol (CH_3_OCH_2_CH_2_OH) to synthesize 0.1 M zinc oxide (ZnO) solution. 0.2 M aluminum oxide (AlO_x_) precursor solution was made by dissolving aluminum chloride (AlCl_3_) (Sigma Aldrich, 99.999%) into a mixed solvent of acetonitrile and ethylene glycol. All the precursor solutions were prepared under an N_2_ environment and stirred at least 2 h to get a transparent solution. A 0.45 μm PTFE filter was used to get the desired precursor solutions.

### Thin film deposition

Aluminum oxide (AlO_x_) thin film was deposited on Mo substrate by spin coating with 2000 rpm for 30 s. The film was then cured at 250 ^o^C on a hot plate for 5 min, and then UV/O_3_ treatment was performed for 5 min. The process repeated twice to get the desired thickness and annealed in a furnace under the atmospheric condition at 350 ^o^C for 2 h. Then, the ZnO semiconductor was deposited by spray pyrolysis at the substrate temperature of 350 ^o^C. The process was repeated to get the desired thickness of semiconductor film. The decomposition of zinc acetate dihydrate in the presence of 2-mythoxyethanol produces zinc hydroxide(Zn(OH)_2_) and acetic acid (CH_3_COOH) follows the reaction:

$${[{\rm{Zn}}({\rm{CH}}}_{3}{{\rm{COO}})}_{2}\cdot 2{{\rm{H}}}_{2}{\rm{O}}]\iff {\rm{Zn}}{({\rm{OH}})}_{2}+2{{\rm{CH}}}_{3}{\rm{COOH}}.$$

Without ammonium acetate (CH_3_COONH_4_), the reaction produces two moles of acetic acid, (2CH_3_COOH) and the decomposition follows to form ZnO, water vapor, carbon dioxide, methane and ketene (CH_2_CO), which can be described by the following reactions:

$${\rm{Zn}}({\rm{OH}})2\to {\rm{ZnO}}+{{\rm{H}}}_{2}{\rm{O}}$$

$${{\rm{CH}}}_{3}{\rm{COOH}}\to {{\rm{CH}}}_{2}{\rm{CO}}\uparrow +{{\rm{H}}}_{2}{\rm{O}}\uparrow $$

$${{\rm{CH}}}_{3}{\rm{COOH}}\to {{\rm{CH}}}_{4}\uparrow +{{\rm{CO}}}_{2}\uparrow $$

Whereas in the presence of ammonium acetate (CH_3_COONH_4_), the reaction produces three moles of acetic acid (3CH_3_COOH), which later decomposes to water vapor, carbon dioxide, methane, ammonia, and ketene and forms ZnO film from the following reaction:

$${\rm{Zn}}{({\rm{OH}})}_{2}\to {\rm{ZnO}}+{{\rm{H}}}_{2}{\rm{O}}\uparrow $$

$${{\rm{CH}}}_{3}{{\rm{COONH}}}_{4}+{{\rm{CH}}}_{3}{{\rm{OCH}}}_{2}{{\rm{CH}}}_{2}{\rm{OH}}\iff {{\rm{CH}}}_{3}{\rm{COOH}}+{{\rm{NH}}}_{4}^{+}+{{\rm{CH}}}_{3}{{\rm{OCH}}}_{2}{{\rm{CH}}}_{2}{{\rm{O}}}^{-}$$

$${{\rm{NH}}}_{4}^{+}+{{\rm{CH}}}_{3}{{\rm{OCH}}}_{2}{{\rm{CH}}}_{2}{{\rm{O}}}^{-}\to {{\rm{NH}}}_{3}\uparrow +{{\rm{CH}}}_{3}{{\rm{OCH}}}_{2}{{\rm{CH}}}_{2}{\rm{OH}}$$

$${{\rm{CH}}}_{3}{\rm{COOH}}\to {{\rm{CH}}}_{2}{\rm{CO}}\uparrow +{{\rm{H}}}_{2}{\rm{O}}\uparrow $$

$$2{{\rm{CH}}}_{3}{\rm{COOH}}\to 2{{\rm{CH}}}_{4}\uparrow +2{{\rm{CO}}}_{2}\uparrow $$

### TFT fabrication

We fabricated the bottom gate, bottom contact ZnO TFT. A 40 nm molybdenum (Mo) was deposited on glass substrate by DC sputtering and patterned for gate electrode. Aluminum oxide (AlO_x_) thin film was deposited on the patterned Mo backplane by spin coating at 2000 rpm for 30 s. After curing for 5 min on a hot plate at 250 ^o^C, UV/O_3_ treatment was performed for 5 min. The process was repeated to get a desired thickness, then annealed in furnace under the atmospheric condition at 350 ^o^C and patterned for via holes. Indium-zinc-oxide (IZO) was deposited by sputtering and patterned for the source/drain electrodes on gate insulator. The ZnO layer was deposited by spray pyrolysis at 350 ^o^C. The distance between the spray nozzle and the substrate was maintained to be about 12 cm. The total time for each cycle of spray deposition step was 60 s.

### Device characterization methods

We characterized the optical and structural properties of the ZnO thin films by measuring UV-visible spectra for transmittance and absorbance (UV-visible spectroscopy), scanning electron microscopy (SEM), atomic force microscopy (AFM), X-ray diffraction (XRD), X-ray photoelectron spectroscopy (XPS), and scanning transmission electron microscope (STEM). To measure the electrical properties of TFTs, Agilent 4156 C semiconductor parameter analyzer was used. The drain voltage (V_DS_) of 0.1 V was used to measure transfer curve by sweeping the gate voltage (V_GS_) from −5 to +5 V. The mobility in the saturation region (V_DS_ ≥ V_GS_ − V_TH_) was obtained by using the Eq. (1),

1 $${{\rm{I}}}_{{\rm{DS}}}=({\rm{W}}/2{\rm{L}}){{\rm{\mu }}}_{{\rm{sat}}}{{\rm{C}}}_{{\rm{i}}}{({{\rm{V}}}_{{\rm{GS}}}-{{\rm{V}}}_{{\rm{TH}}})}^{2},$$

where I_DS_, V_GS_, μ_sat_, W, L, V_TH_, and C_i_, are the drain current, applied gate voltage, saturation mobility, channel width, channel length, threshold voltage, and gate insulator capacitance, respectively. The V_TH_ was determined from the x-axis intercept of the √(I_DS_) versus V_GS_ plot by linear extrapolation. The saturation mobility calculated from the linear part of the √(I_DS_) vs V_GS_ curve. The subthreshold swing (SS) obtained from the linear region of the log (I_DS_) versus V_GS_ fit by using the Eq. (2),

2 $${\rm{SS}}={{\rm{dV}}}_{{\rm{GS}}}/{\rm{d}}(\log \,{{\rm{I}}}_{{\rm{DS}}}).$$

## Supplementary information

Supplementary information.
