# Supplementary material for: Significant improvement of spray pyrolyzed ZnO thin film by precursor optimization for high mobility thin film transistors
Source: Sci Rep. 2020 Jun 2;10:8999. doi: 10.1038/s41598-020-65938-6 (PMC7265479; doi:10.1038/s41598-020-65938-6)
Supplement: Supplementary file 1 — Supplementary information. [file 41598_2020_65938_MOESM1_ESM.docx]

**Supporting Information**

**Significant improvement of spray pyrolyzed ZnO thin film by precursor optimization for high mobility thin film transistors**

Jewel Kumer Saha, Ravindra Naik Bukke, Narendra Naik Mude, and Jin Jang *

Advanced Display Research Center (ADRC), Department of Information Display, Kyung Hee University, 26, Kyungheedae-ro, Dongdaemun-gu, Seoul 02447, Korea
E-mail: jjang@khu.ac.kr

**
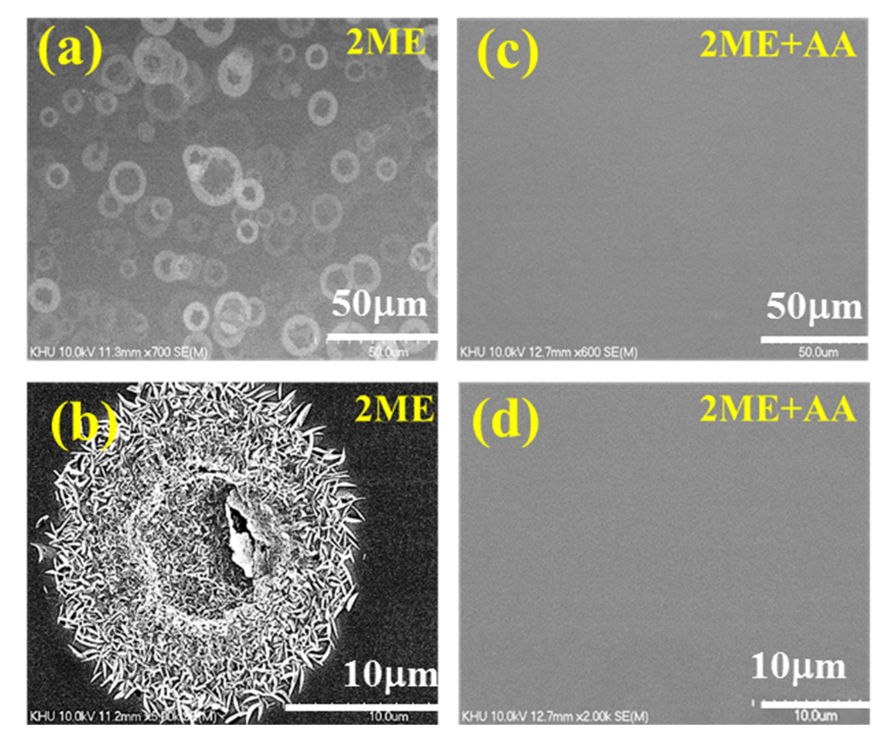
**

**Figure S1** SEM image of ZnO thin film deposited at 350 ^o^C using 2 methoxy-
ethanol (2ME) as a precursor solvent **(a-b)** without and **(c-d)** with AA.

**
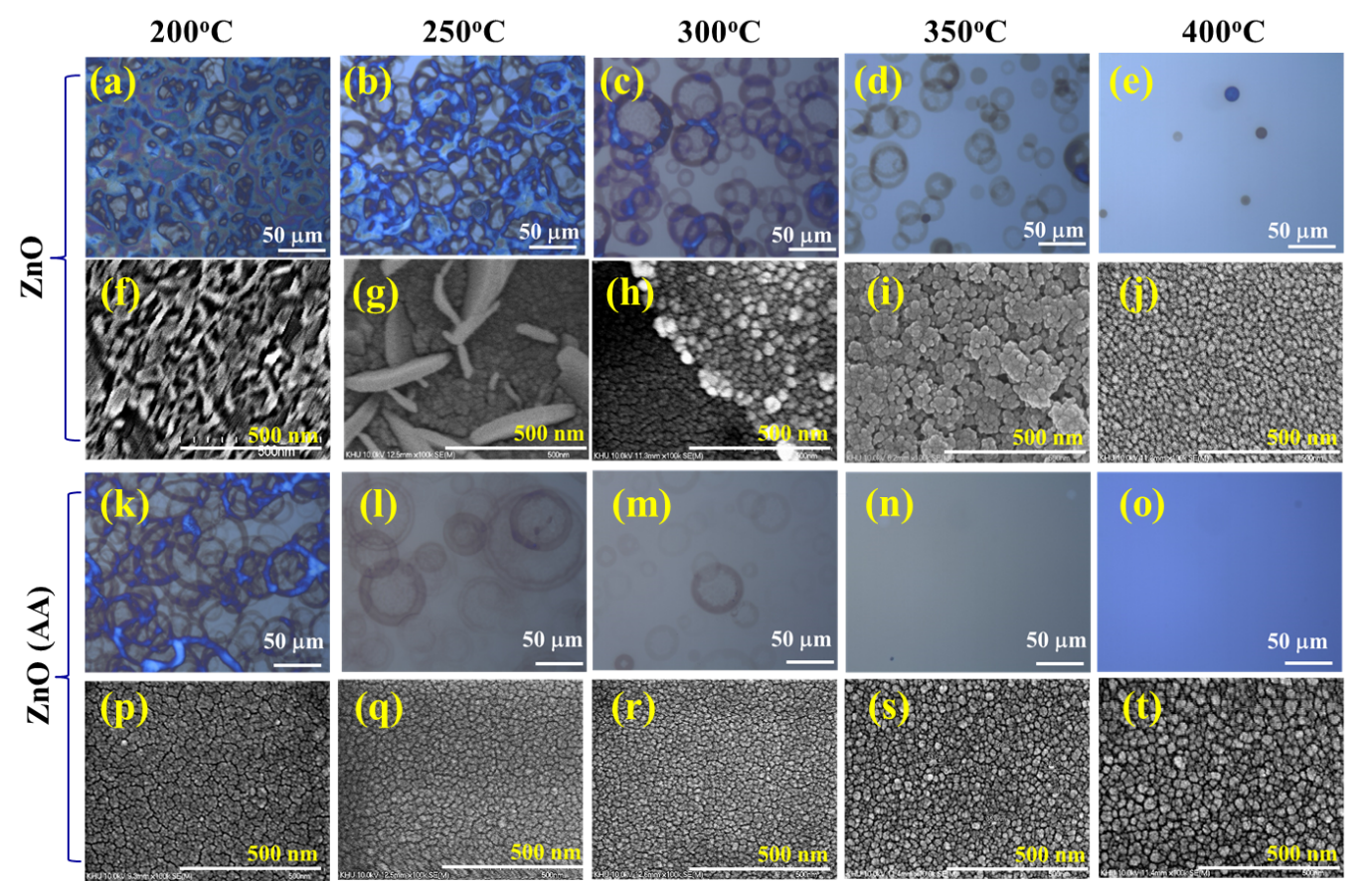
**

**Figure S2 (a-e)** Optical microscopy images and **(f-j)** SEM images of ZnO thin films deposited at 200 to 400 ^o^C without AA. **(k-o)** Optical microscopy images and **(p-t)** SEM images of ZnO thin films deposited at 200 to 400 ^o^C without AA. The amount of bubbles reduces with both increasing the temperature and adding AA in the precursor solution.


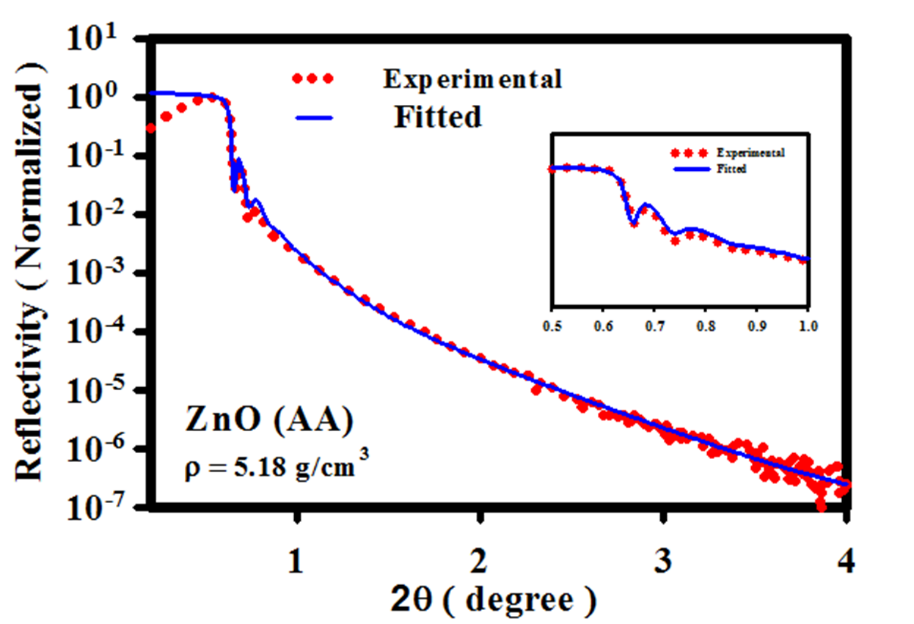


**Figure S3** XRR spectra for the ZnO thin film with AA.


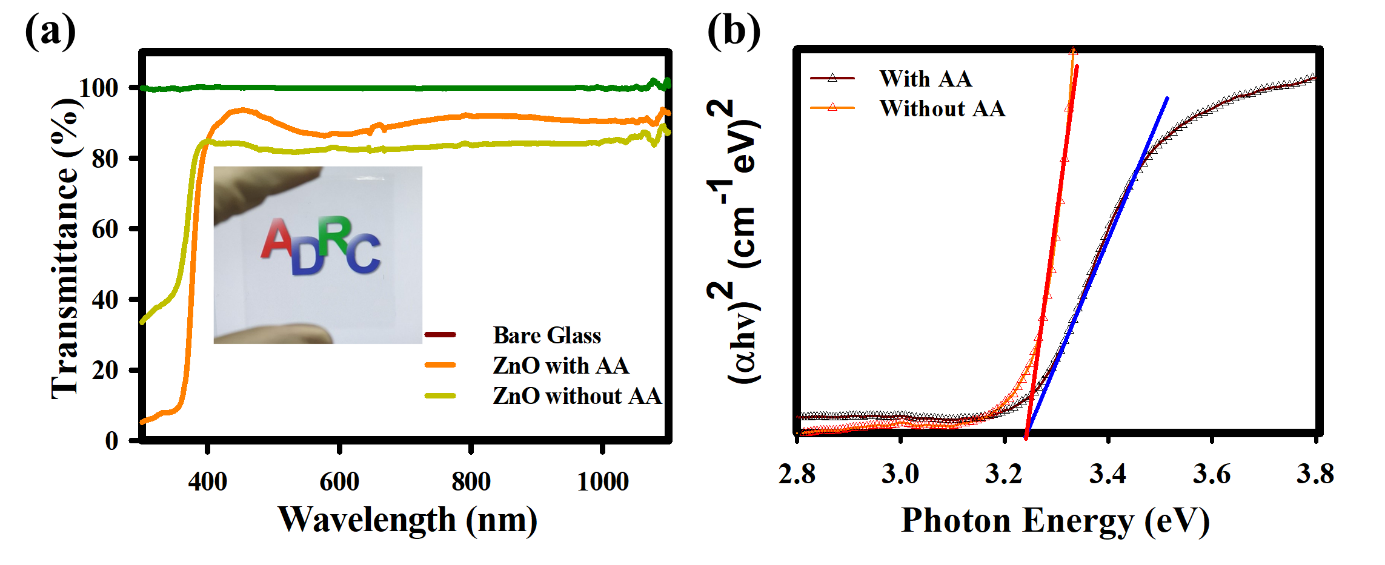


**Figure S4 (a)** UV –Vis transmittance spectra of ZnO thin film deposited on glass substrate, the photograph of the ADRC (Advanced Display Research Center) logo under the ZnO thin film is shown in inset. The transmittance increases from 84 to 90% when AA is added, **(b)** The optical band gap (extracted using Tauc plots) of ZnO thin films without and with AA are similar ~3.25 eV.


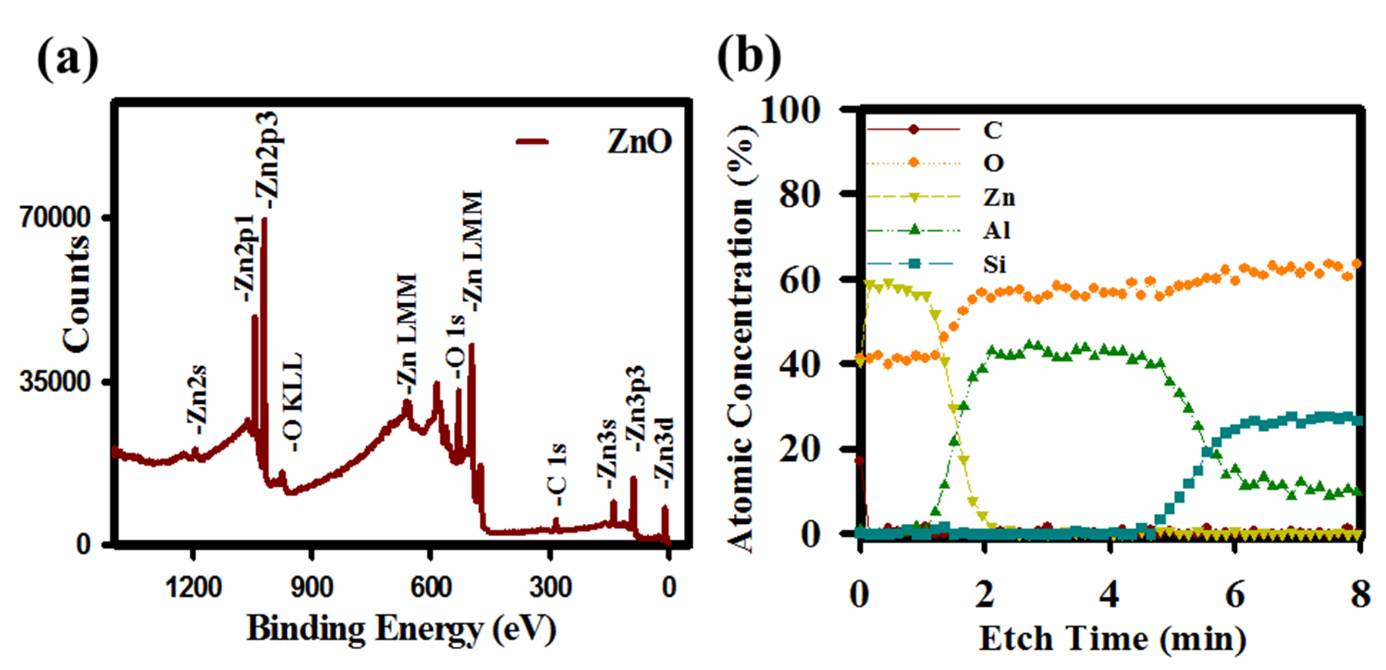


**Figure S5 (a)** XPS spectrum of ZnO film **(b)** depth profile of ZnO film on Al_2_O_3_ film


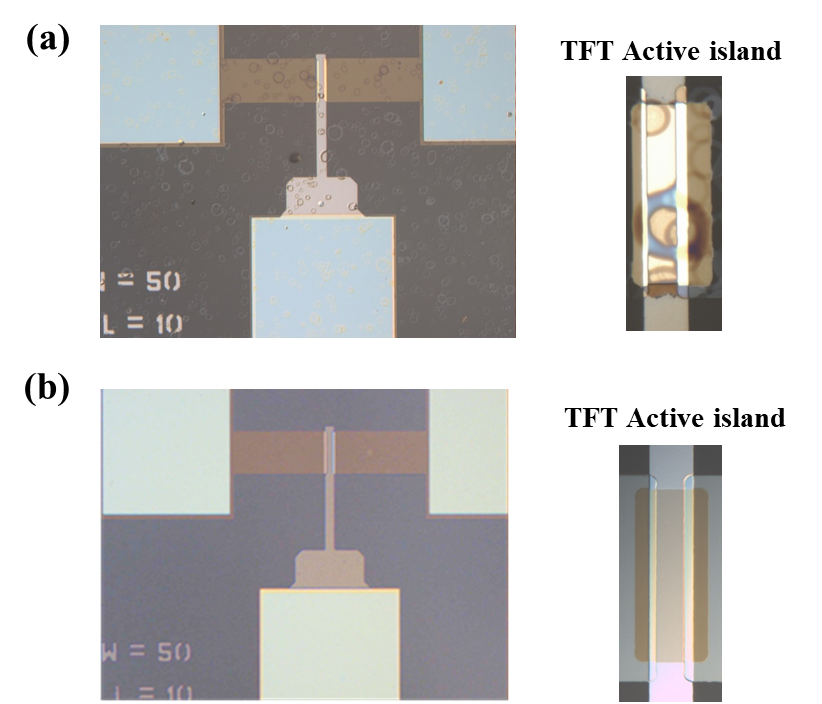


**Figure S6** Optical images of the ZnO TFT **(a)** without addition of AA, and **(b)** with AA in the precursor, respectively.


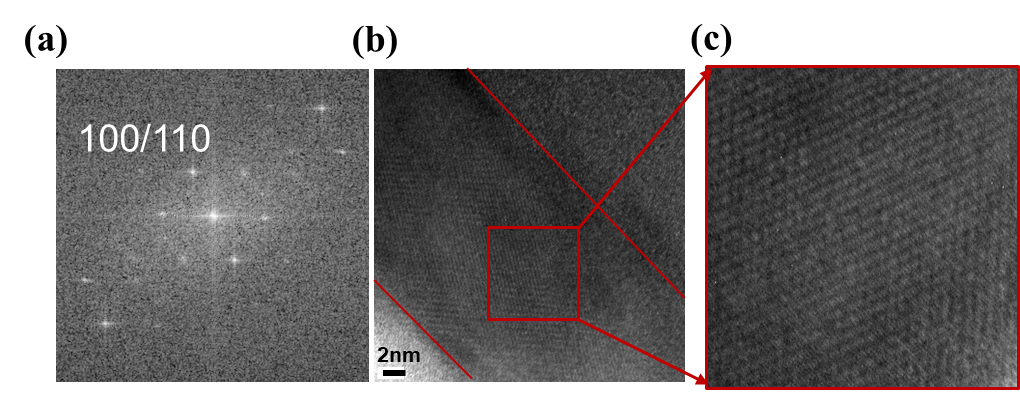


**Figure S7** Transmission electron microscopy (TEM) image showing the crystallinity of ZnO film. **(a)** Fast Fourier transform (FFT) – **(b)** large view HRTEM cross-section image and **(c)** details of the active layer seen perpendicularly from the c-axis


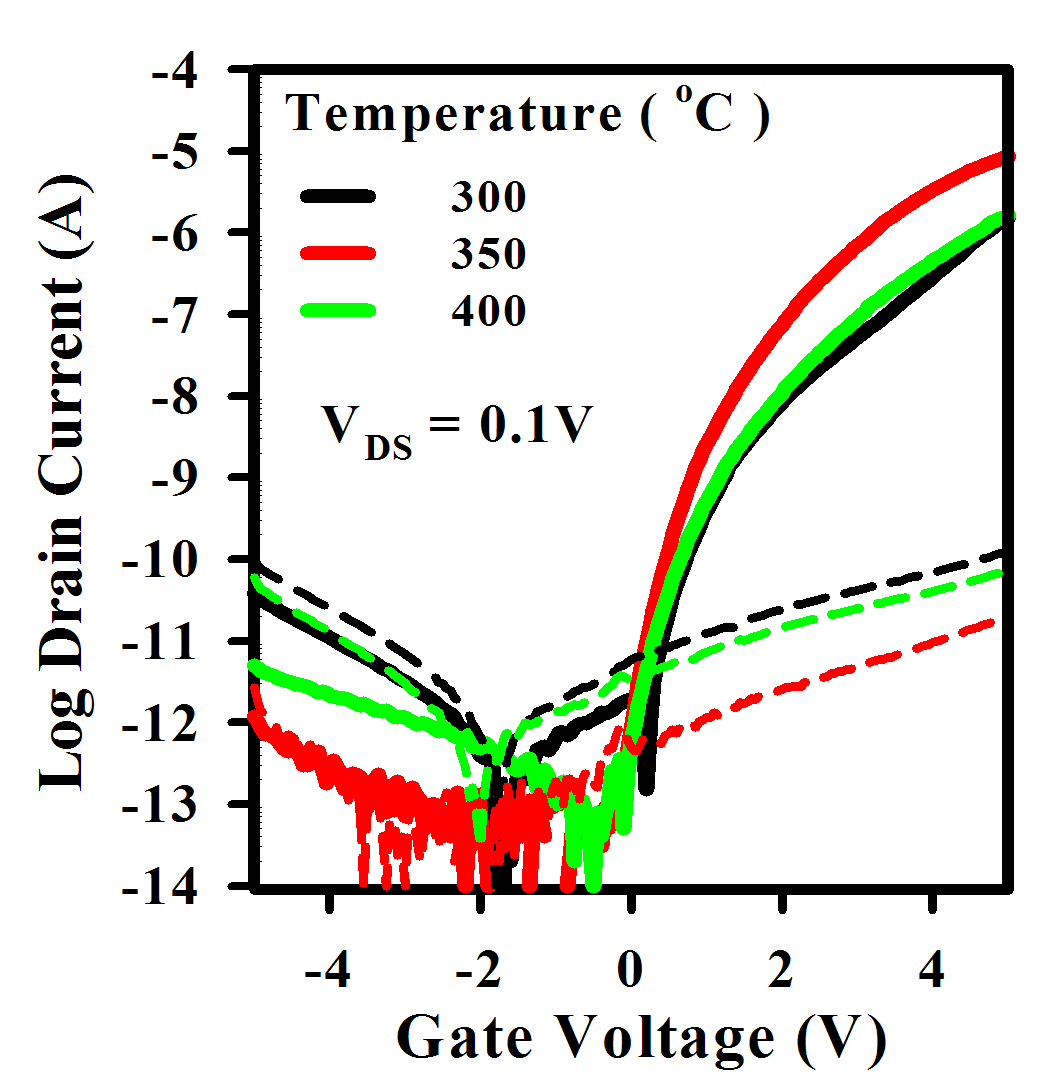


**Figure S8** Transfer curves of ZnO TFTs fabricated by spray pyrolysis from 300 to
400 ^o^C. The transfer curve of the TFT was measured by sweeping V_GS_ from -5 to +5 V at the V_DS_ of 0.1 V.
